# Genetic investigation of hydrogenases in *Thermoanaerobacterium thermosaccharolyticum* suggests that redox balance via hydrogen cycling enables high ethanol yield

**DOI:** 10.1128/aem.01109-24

**Published:** 2025-01-10

**Authors:** Layse C. de Souza, Christopher D. Herring, Lee R. Lynd

**Affiliations:** 1 Centro de Engenharia Genética e Biologia Molecular (CBMEG), Universidade Estadual de Campinas (UNICAMP) 196609 , Campinas, São Paulo, Brazil; 2 Programa de Pós-Graduação em Genética e Biologia Molecular, Universidade Estadual de Campinas Instituto de Biologia 124594 , Campinas, São Paulo, Brazil; 3 Terragia Biofuel, Hanover, New Hampshire, USA; 4 Thayer School of Engineering, Dartmouth College 145792 , Hanover, New Hampshire, USA; 5 Center for Bioenergy Innovation, Oak Ridge National Laboratory 6146 , Oak Ridge, Tennessee, USA; University of Nebraska-Lincoln, Lincoln, Nebraska, USA

**Keywords:** *T. thermosaccharolyticum*, electron flow, redox equilibrium, ferredoxin:NADP^+^oxidoreductase, ferredoxin recycling, genetic manipulation

## Abstract

*Thermoanaerobacterium thermosaccharolyticum* is an anaerobic and thermophilic bacterium that has been genetically engineered for ethanol production at very high yields. However, the underlying reactions responsible for electron flow, redox equilibrium, and how they relate to ethanol production in this microbe are not fully elucidated. Therefore, we performed a series of genetic manipulations to investigate the contribution of hydrogenase genes to high ethanol yield, generating evidence for the importance of hydrogen-reacting enzymes in ethanol production. Our results indicate that a high ethanol yield, >85% of the theoretical maximum, only occurs when the *hfsD, hydAB*, and *nfnAB* genes are all present together, while the *hfsB* gene is absent. We propose that the products of these three gene clusters facilitate an NADPH-generating reaction via hydrogen cycling, with a stoichiometry comparable with a canonical ferredoxin:NADP^+^ oxidoreductase (FNOR; EC 1.18.1.2) reaction. The hypothesized mechanism provides a balance of nicotinamide cofactors and facilitates ferredoxin recycling, leading to progress in optimizing the energy conversion of biomass-derived sugars to ethanol.

**IMPORTANCE:**

Our study elucidates the crucial role of electron flow and redox balancing mechanisms in improving ethanol yields from renewable biomass. We delve into the mechanism of electron transfer, highlighting the potential of key genes to be leveraged for enhanced ethanol production in anaerobic microbial species. We suggest by genetic investigation the existence of a novel Ferredoxin:NADP+ Oxidoreductase (FNOR) reaction, comprising HfsD, HydAB, and NfnAB enzymes, as a promising avenue for achieving balanced stoichiometry and efficient ethanol synthesis. Our findings not only advance the understanding of microbial metabolism but also offer practical insights for developing strategies to improve bioenergy production and sustainability.

## INTRODUCTION

*Thermoanaerobacterium thermosaccharolyticum* is an anaerobic and thermophilic bacterium capable of fermenting a wide range of biomass-derived sugars and polymers into ethanol, acetate, lactate, formate, and butanol. Its xylan- and hemicellulose-conversion capabilities are particularly attractive for bioenergy production (1, 2). *T. thermosaccharolyticum* is similar to *Thermoanaerobacterium saccharolyticum* but has important differences, especially in terms of butanol production and a higher pH range in *T. thermosaccharolyticum* (3). The wild-type *T. thermosaccharolyticum* has been extensively studied, offering valuable insights into the genetic mechanisms underlying its ethanol production pathway. A review from Olson, Sparling, and Lynd (4) provides a comprehensive representation of the current understanding of ethanol metabolism in *Thermoanaerobacterium* spp*.,* highlighting key metabolic pathways. Although considerable advancements have been made in directing flux to ethanol at >85% yield (herein referred to as the ethanologen phenotype) and achieving a titer of 70 g/L (5), the reactions responsible for electron flow, redox balancing, and their role in ethanol production in this microbe are not fully understood.

The first successful efforts to increase ethanol yield in *Thermoanaerobacterium* spp. focused on deleting enzymes responsible for directing carbon flow to acetate and lactate (6). Later work investigated the role of hydrogenases and the elimination of hydrogenase activity as an electron-centric strategy to direct flux (7). Balanced electron flow is essential for modulating redox homeostasis while producing a desired product. By directing the flow of electrons to protons or receiving them from dihydrogen, hydrogenases can play an important role in anaerobic bacteria (8, 9). Depending on the thermodynamics of the reaction and the prevailing conditions, these enzymes can either expel excess reducing power through the reduction of protons or serve as an electron source by oxidizing molecular hydrogen.

In wild-type strains, acetate production is associated with high hydrogenase activity, as this enables the recycling of ferredoxin without generating any nicotinamide cofactors, which are not needed for acetate synthesis. In contrast, low hydrogenase activity is associated with ethanol synthesis, as reduced nicotinamide cofactors are consumed in the final two steps of ethanol production (7, 10, 11).

*T. thermosaccharolyticum* harbors three different hydrogenase clusters: *ech*, an energy-conserving hydrogenase that is typically inactive in wild-type *T. saccharolyticum* strains; *hydABCD,* an electron-bifurcating hydrogenase that facilitates energy conversion by coupling exergonic and endergonic reactions; and the *hfsABCD* operon, a ferredoxin-linked hydrogenase (7).

The *hfsABCD* operon was identified in *T. saccharolyticum* as encoding the main hydrogenase responsible for transferring electrons to protons, generated from the sugar-to-ethanol pathway. The *hfs* operon is composed of four distinct genes: *hfsA, hfsB, hfsC,* and *hfsD* (7). The sequence of the HfsA protein does not align well with any known protein; however, part of its structure matches the large subunits of [FeFe] hydrogenases. The HfsB protein is a [FeFe]-hydrogenase with a “PAS”' domain that may act as a sensory domain, responding to changes in the redox potential within the cell. The activity of this protein has been inferred through homology studies that compared the *hfs* operon from *T. saccharolyticum* with the hydrogenases found in *Ruminococcus albus*. In *R. albus,* the protein HydS, which has the same sensory domain as HfsB, is required for growth at high molecular hydrogen partial pressure. The HfsC protein has homology with serine phosphatase proteins, and it is predicted to control physiological aspects of metabolism. Finally, HfsD is a ferredoxin-linked protein that has high similarity to the HydA2 hydrogenase from *R. albus* (7, 10). Deletion of the *hfsABCD* operon not only reduces hydrogen and acetate production but also affects growth rate. Surprisingly, either point mutations or deletion of the *hfsB* subunit of this operon led to high ethanol yield and low acetate production without affecting the growth rate of the cells (12, 13). It was hypothesized that *hfsB* encodes a regulator of other genes in this operon. Moreover, the deletion of *hfsC* and *hfsD* resulted in a diversion of flux toward lactate production, suggesting inadequate functioning of the pyruvate:ferredoxin oxidoreductase (PFOR) reaction (13), which converts pyruvate to acetyl-CoA along with the formation of reduced ferredoxin.

To account for electron flux in fermentative anaerobes, it is important to consider the overall balance of reduced nicotinamide cofactors involved in glycolysis and ethanol formation. In *T. thermosaccharolyticum* glycolysis, two NADH are produced by glyceraldehyde-3-phosphate dehydrogenase (GAPDH) per glucose. Restated, one NADH is produced by GAPDH per half-glucose. In the terminal steps of ethanol production, two NAD(P)H are consumed per half-glucose; therefore, another NAD(P)H-evolving reaction must occur. The PFOR reaction transfers electrons to ferredoxin, and those electrons must be transferred somewhere to regenerate oxidized ferredoxin for the further activity of PFOR. However, the fate of those electrons and the enzymes involved is unclear. One possible candidate could be the NADH-dependent reduced ferredoxin:NADP^+^ oxidoreductase (Nfn), a bifurcating NADPH-linked enzyme that has been demonstrated to be essential for ethanol production in *T. saccharolyticum*. Its absence is responsible for a drastic reduction in ethanol yield (14). However, the reaction encoded by the NfnAB complex (2 NADP^+^ + NADH + Fd_red_ → 2 NADPH + NAD^+^ + F_ox_) does not enable balanced stoichiometry. Although the NfnAB complex consumes the NADH generated from glycolysis, the two NADPH it generates are incompatible with the two-step conversion of acetyl-CoA to ethanol: the conversion of acetaldehyde to ethanol (the ADH reaction) can proceed with either NADH or NADPH, whereas the conversion of acetyl-CoA to acetaldehyde (the ALDH reaction) requires NADH (15). Therefore, the combined reaction catalyzed by AdhE, a bifunctional enzyme that facilitates both the ALDH and ADH reactions, can use either 1 NADH + 1 NADPH or 2 NADH. Although capable of recycling ferredoxin while producing nicotinamide cofactors, the gene *nfnAB*, by itself, cannot be responsible for balancing the reaction when considering the cofactor specificity of the ALDH and ADH reactions (15). To balance glycolysis and ethanol production, another ferredoxin-regenerating reaction must occur.

Considering the need for both NADH and NADPH for ethanol synthesis, it is thought that a ferredoxin:NAD(P)^+^ oxidoreductase (FNOR; EC 1.18.1.2) reaction could be the missing element responsible for promoting a balanced stoichiometry for ethanol production at high yield. From reduced ferredoxin, the hypothetical FNOR reaction converts NAD^+^ to NADH (Fd_red_ + NAD^+^ + H^+^ → Fd_ox_ + NADH). However, a direct transfer from reduced ferredoxin to NAD^+^ is energetically wasteful, given the high negative redox potential (E°') of ferredoxin relative to NAD(P)H, unless it is coupled to another reaction (16, 17). Due to the mismatch in redox potentials between ferredoxin and NAD(P)H, a direct transfer would result in a loss of energy. A wide range of anaerobes overcome this thermodynamic challenge and conserve energy by using bifurcating hydrogenases (18). *Thermotoga maritima* is an example of an organism that couples endergonic reductions with highly exergonic reactions using the bifurcating Hyd complex (19).

The identity of the hypothesized FNOR in *T. thermosaccharolyticum* has been an ongoing mystery. Previous studies identified the protein Tsac_1705 as being responsible for the FNOR reaction (16). However, its genomic location strongly indicates its association with the pyrimidine synthesis pathway rather than the ethanol pathway of *Thermoanaerobacterium* spp. We hypothesize that the ferredoxin:NADP^+^ oxidoreductase reaction in this microorganism is not the result of a single component. The purification of all the components of a multi-protein reaction complex, along with the demonstration of its mechanistic pathway, can be challenging. Therefore, we sought to use a genetic approach to begin unraveling this complex situation by identifying the components involved.

In this study, we investigate the importance of hydrogenases for ethanol production in *T. thermosaccharolyticum,* presenting genetic evidence for the necessity of the *hfsD, hydAB,* and *nfnAB* clusters for high ethanol yields. We hypothesize that the products of these gene clusters could create a functional NADPH-generating reaction equivalent to a canonical FNOR through hydrogen cycling, providing nicotinamide cofactor balance and recycling of ferredoxin.

## RESULTS

The idea of a possible relationship between the *hyd* and *hfs* operons arose after an intriguing observation in *T. thermosaccharolytium*. Although the deletion of the *hfsB* gene was previously demonstrated as a method to obtain ethanologen strains in *T. saccharolyticum* (13), we observed that the phenotype resulting from the *hfsB* deletion was not stable. After a few transfers, the strain reverted from the ethanologen phenotype to a mixed acid production phenotype, decreasing the theoretical ethanol yield by 34% (Table 1; Table S1). Whole-genome sequencing analysis revealed a mutation in the *hydC* gene (Table 2; Table S1). These results suggest that the ethanologen phenotype is influenced not only by the presence of the *hfs* genes but also by the genes in the *hyd* operon, raising the possibility that the encoded hydrogenases may both have an impact on the ethanol production pathway.

**TABLE 1:** Fermentation profile comparison between *∆hfsB* strain LL1862 before and after serial transfers^*a*^

Strain	Consumed sugars (mM)	Formate (mM)	Acetate (mM)	Ethanol (mM)	% Theoretical maximum yield of ethanol
LL1862 - *∆hfsB* before serial transfer	10.41 ± 0.66	1.30 ± 0.09	3.17 ± 0.29	39.27 ± 0.46	98.66% ± 0.06%
LL1862 - *∆hfsB* after serial transfer	13.04 ± 0.00	4.56 ± 0.06	11.25 ± 0.01	31.94 ± 0.04	64.48% ± 0.02%

^*a*^ Data represent averages of the results of replicates *n* ≧ 3. Strains were fermented in CTFUD with 5 g/L cellobiose. Values are presented as mean ± standard deviation.

**TABLE 2:** Mutations found in *∆hfsB* strain LL1862 after serial transfer

Genome region	Gene locus	Gene description	Type	Reference	Allele
1062443	THETHE_RS05140	Ferredoxin - *hyd* operon subunit C	Deletion	T	−^*b*^
915636-915973	THETHE_RS04425	Nucleotidyltransferase	2 Insertions	−	AA
383054-383138	THETHE_RS01860	Elongation factor Tu	3 SNVs^*a*^	−	−
646753	THETHE_RS03135	Proline reductase	SNV	C	A
1773456	THETHE_RS08760	DNA-binding response regulator	SNV	T	C
1898491	THETHE_RS09355	LacI family transcriptional regulator	SNV	C	A

^*a*^ SNV, single nucleotide variant.

^*b*^ −, no data.

**Fig 1:**
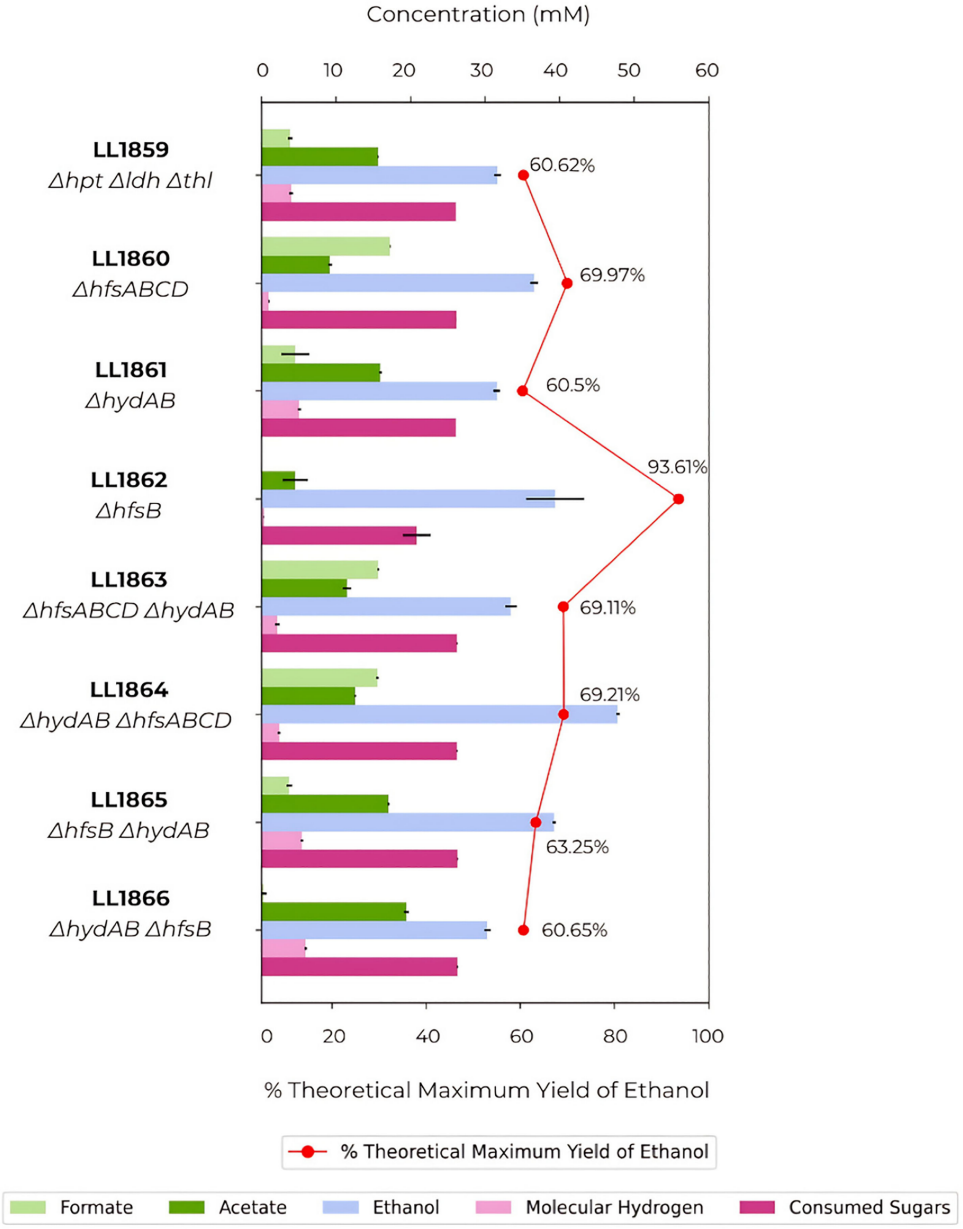
Fermentation profile after knockout of the *hfs* operon, *hydAB* and *hfsB* genes, individually or in combination. Strains were fermented in CTFUD medium with 0.5 g/L of yeast extract and 5 g/L of cellobiose. Data represent averages of the results of replicates N ≧ 3.

To test this idea, we deleted the *hfs* and *hyd* genes both separately and in combination. Complete deletion of the *hfs* operon (strain LL1860) and deletion of *hydAB* (strain LL1861) resulted in a mixed acid fermentation. The ethanologen phenotype was observed only in strain LL1862, in which both *hfsD* and *hydAB* are present while *hfsB* is absent (Fig. 1), which is consistent with Eminoglu’s findings in *T. saccharolyticum* (13). This strain exhibited increased ethanol yield and decreased acetate and formate production relative to the parent strain; however, when the *hydAB* genes were also deleted (in strain LL1865), there was a shift in the fermentation profile. It exhibited not only increased formate and acetate levels, but the ethanol yield decreased by almost 30%. None of the strains in which genes from the *hfs* operon and *hydAB* were deleted at the same time showed high ethanol yield (Fig. 1). Although final ethanol concentrations varied between strains, the relationship between genotype and phenotype is clear when also considering sugars consumed and ethanol yield.

**Fig 2:**
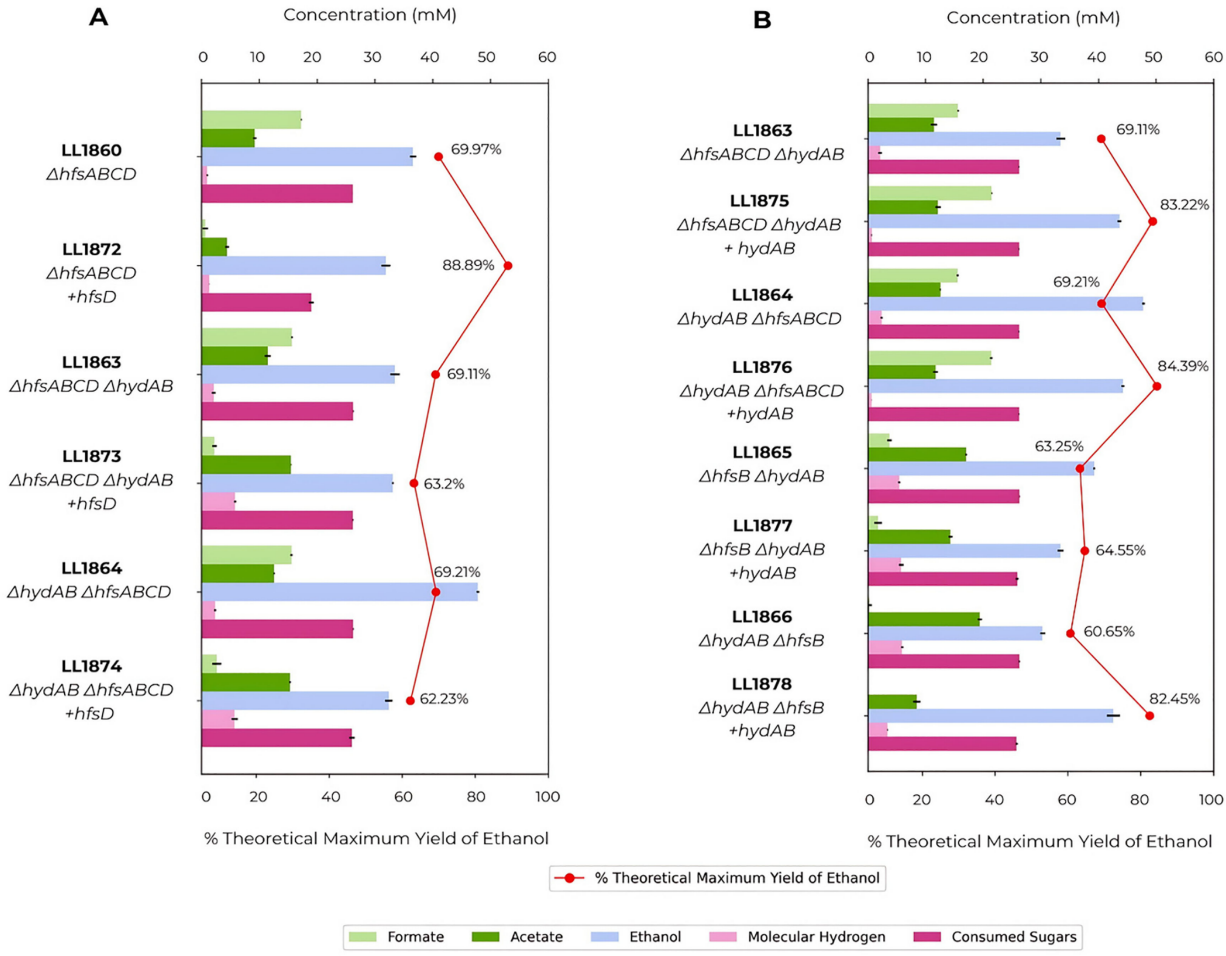
Comparison of the fermentation profile of strains after overexpression of (A) *hfsD* or (B) *hydAB* genes. Strains were fermented in CTFUD with 0.5 g/L of yeast extract and 5 g/L of cellobiose. Data represent averages of the results of replicates N ≧ 3.

Based on previous results with the *hfs* genes (12, 13), we hypothesized that high ethanol yield in *Thermoanaerobacterium* spp. requires not only the inactivation of *hfsB* but also the presence of *hfsD*, which encodes a ferredoxin-linked [FeFe] hydrogenase. To test whether the ethanologen phenotype requires the simultaneous presence of both *hfsD* and *hydAB* while *hfsB* is absent, we produced new strains and compared their fermentation profiles (Fig. 2). First, we overexpressed the *hfsD* gene in strain LL1860 (*ΔhfsABCD*), creating the strain LL1872. This manipulation resulted in an ethanologen phenotype comparable with the ethanol profile of strains in which the *hfsB* gene is deleted alone (LL1862, Fig. 1). Reintroducing *hfsD* in strains with the *hydAB* deletion did not increase ethanol yield for the strains LL1873 and LL1874*,* reinforcing the hypothesis that both *hfsD* and *hydAB* are indispensable for achieving high ethanol production. The reintroduction and overexpression of *hydAB* genes do not show clear and conclusive results, possibly due to the instability of *hfsB* knockout strains noted previously and the background activity of the *ech* operon (Fig. 2). Although they share the same genetic modifications, the order of the manipulations seemed to affect the phenotype in strains LL1877 and LL1878. The latter showed a larger increase in ethanol yield after the introduction of the *hydAB* gene. We speculate this could be due to the more recent deletion of the *hfsB* gene, allowing less time for spontaneous mutations.

Interestingly, the absence of the *hfsD* gene seems to be associated with higher formate production. Strains LL1860, LL1863, and LL1864 had formate production four times higher than the parent strain (Fig. 1 and 2). In contrast, in ethanologen strains where *hfsD* was present in the absence of *hfsB* (LL1862 and LL1872), formate production was minimal (Fig. 1 and 2).

**Fig 3:**
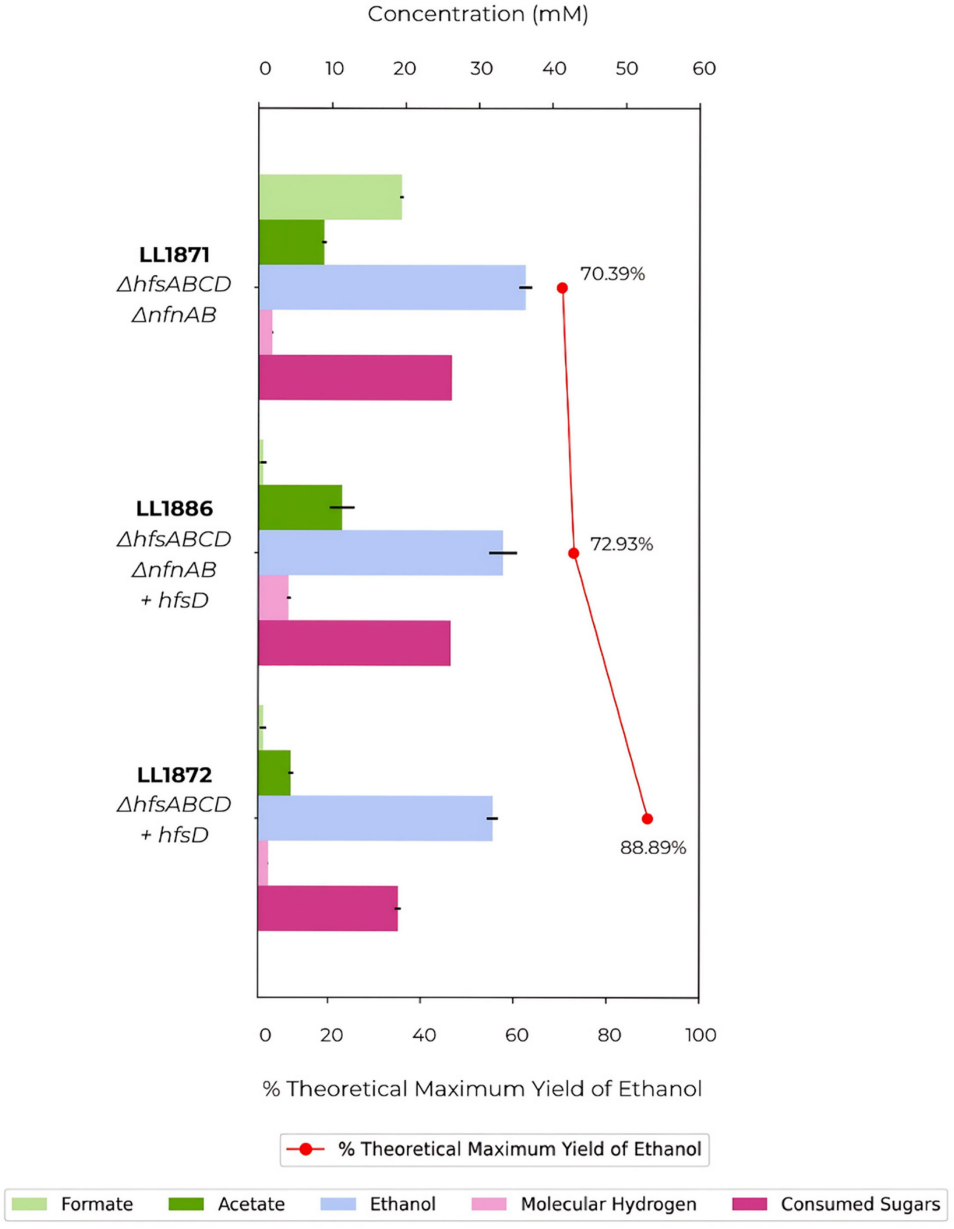
Comparison of the fermentation profile between an ethanologen strain and the effect of the deletion of *nfnAB* on this strain. Strains were fermented in CTFUD with 0.5 g/L of yeast extract and 5 g/L of cellobiose. Data represent averages of the results of replicates *N* ≧ 3.

To determine if *nfnAB* is also important for high ethanol yield, we deleted it from the ethanologen strain LL1872, creating strain LL1889 (Fig. 3). We observed a decrease in ethanol production when *nfnAB* was absent, which is consistent with previous studies indicating the need for *nfnAB* for ethanol production (14). We then constructed a plasmid carrying both *hfsD* and *nfnAB*, aiming to assess whether the expression of these genes in LL1860 (*∆hfsABCD*) would be sufficient to restore the ethanologen phenotype. Unfortunately, the plasmid seemed to exhibit toxicity in *Escherichia coli*, preventing successful cloning before transformation into *T. thermosaccharolyticum*. Multiple attempts were made with consistently unfavorable outcomes.

A previous study in *T. saccharolyticum* showed that the *hfsABCD* and *hydABCD* operons are the only active hydrogenases (7). Consequently, our expectation was minimal or no H_2_ production when both operons were deleted in *T. thermosaccharolyticum*. However, H_2_ production was observed in the *hfsABCD* and *hydAB* knockouts (LL1863 and LL1864 in Fig. 1) . Besides the *hfs* and *hyd* operons, both species have a third gene cluster that encodes for the energy-conserving hydrogenase *ech*, previously thought to be inactive in *T. saccharolyticum* (7). We hypothesized that *ech* is either more active in *T. thermosaccharolyticum* or is activated in the context of the *hfs hyd* double mutants. To test this, we deleted it in *T. thermosaccharolyticum* double mutants to generate hydrogenase-minus strains (*Δhfs Δhyd Δech;* LL1868 and LL1869), which showed no hydrogen production (Table 3). The triple mutants were unable to grow well in media with low concentrations of yeast extract but grew normally in CTFUD with 4.5 g/L of yeast extract.

**TABLE 3:** Fermentation profile after deletion of the *ech* operon^*a*^

Sample	Genotype	Remaining sugars (mM)	Formate (mM)	Acetate (mM)	Ethanol (mM)	Molecular hydrogen (mM)	% Theoretical maximum yield of ethanol
LL1867	*∆ech*	0.21 ± 0.00	4.04 ± 0.16	16.68 ± 0.20	33.50 ± 0.19	5.07 ± 0.17	64.13% ± 0.00%
LL1868	*∆hfsABCD ∆hydAB ∆ech*	0.16 ± 0.16	20.11 ± 2.24	16.16 ± 7.28	47.87 ± 0.42	0.01 ± 0.00	92.13% ± 0.01%
LL1869	*∆hydAB ∆hfsABCD ∆ech*	3.17 ± 0.29	20.52 ± 0.20	7.79 ± 0.06	39.11 ± 1.64	0 ± 0.01	84.43% ± 0.03%

^*a*^ Data represent averages of the results of replicates *n* ≧ 3. Strains were grown in 5 g/L cellobiose.

All fermentation data for the strains used in this work, as well as the carbon balance and electron balance, can be found in Table S2.

To better evaluate the correlation between the observed phenotypic reversion and the occurrence of spontaneous mutations in hydrogenase genes such as *hyd,* we isolated and sequenced multiple independent isolates. A total of 10 individual colonies of a freshly created *hfsB* knockout strain equivalent to LL1862*,* all initially showing high ethanol yield, were serially transferred for 10 days, after which they displayed a mixed acid fermentation profile (Table S3). Subsequently, whole-genome sequencing was conducted. Mutations identified in the *hyd* operon are described in Table 4, whereas other significant mutations are listed in Tables S4 and S5. Of the 10 transfers analyzed, eight strains had mutations in the *hyd* operon. Strains exhibiting reverted phenotype, RF2 and RF4, showed a deletion of 24 base pairs in the *hydB* gene at identical genomic positions. Strain RF6 and RF9 showed an identical point mutation in *hydA*. Strains RF5, RF7, RF8, and RF10 showed the presence of insertion sequence (IS) elements and small genetic inversions, but the analysis suggests four different genetic events. Strains RF1 and RF3 had no mutation in the hydrogenase genes. However, these strains had mutations in THETHE_RS12345, RNA polymerase recycling motor HelD, and THETHE_RS13365, a single-stranded DNA-binding protein (Tables S4 and S5). The same genes acquired independent mutations in other strains, suggesting a biological connection, possibly at the level of gene transcription.

**TABLE 4:** Description of *hyd* operon mutations in the 10 strains exhibiting reverted phenotype (RF) after 10 serial transfers

Strain	Gene locus	Gene description	Genomic findings	Position
RF1	−^*a*^	−	No *hyd* mutation	−
RF2	THETHE_RS05155	NADH:ubiquinone oxidoreductase NADH-binding - *hyd* operon subunit B	Deletion	1064167-1064190
RF3	−	−	No *hyd* mutation	−
RF4	THETHE_RS05155	NADH:ubiquinone oxidoreductase NADH-binding - *hyd* operon subunit B	Deletion	1064167-1064190
RF5	THETHE_RS05160	Hydrogenase Fe-only - *hydA* operon subunit A	Inversion and IS insertion	1065493-1065589
RF6	THETHE_RS05160	Hydrogenase Fe-only - *hydA* operon subunit A	Deletion	1066786
RF7	THETHE_RS05160	Hydrogenase Fe-only - *hydA* operon subunit A	Inversion and IS insertion	1065457-1065602
RF8	THETHE_RS05160	Hydrogenase Fe-only - *hydA* operon subunit A	Inversion and IS insertion	1065677-1065809
RF9	THETHE_RS05160	Hydrogenase Fe-only - *hydA* operon subunit A	Deletion	1066786
RF10	THETHE_RS05160	Hydrogenase Fe-only - *hydA* operon subunit A	Inversion and IS insertion	1065485-1065576

^*a*^ −, no data.

## DISCUSSION

Since ethanol production in *Thermoanaerobacterium* spp. is regulated to a great extent by electron availability (20), an account of the electron balance in the fermentation pathway has been of great interest. To achieve high ethanol yields, it is crucial to understand the electron flow and which genes are involved in it.

Existing literature indicates that the presence of *hfsD* and the absence of *hfsB* are a key feature for high-yield ethanol production in *Thermoanaerobacterium* spp. In *Ruminococcus albus*, the hydrogen-sensing hydrogenase HydS appears to regulate the biosynthesis of HydA2, a ferredoxin-dependent FeFe hydrogenase. Interestingly, *hydA2* and *hydS* are arranged in a comparable configuration to *hfsD* and *hfsB* within the *hfs* operon. Additionally, HydA2 and HydS are known to be functionally similar to HfsD and HfsB, respectively. Besides these enzymes, *R. albus* also carries a third hydrogenase cluster, *hydABC* (10), but the interplay among these three enzymes is not yet fully understood. In *T. thermosaccharolyticum*, our observation of spontaneous mutations in *hydAB* and the subsequent reversion to a mixed acid phenotype strongly indicate their importance for the ethanologen phenotype. Similarly, extensive studies have been conducted on the role of *nfnAB,* showing its importance for ethanol synthesis (14).

In this study, we provide evidence that *hfsD*, *hydAB,* and *nfnAB* are concurrently essential for the ethanologen phenotype previously documented in *T. saccharolyticum*. We hypothesize that the products of the three gene clusters are acting in conjunction with each other to some extent and might create a reaction with a stoichiometry similar to a canonical FNOR, with H_2_ as a key intermediate. The FNOR reaction converts NAD(P)^+^ to NAD(P)H using reduced ferredoxin. However, the direct transfer from ferredoxin to NAD(P)^+^ is thermodynamically wasteful, as is the transfer of electrons to protons forming H_2_ (17). Efficiency therefore depends on coupling reactions. Electron-bifurcating hydrogenases such as HydABCD, are able to couple reduction of low-potential electron acceptors (NAD^+^ and Fd_ox_) with highly exergonic reactions (oxidation of H_2_), promoting energy conservation. However, under high H_2_ partial pressure, this reaction can shift toward the reverse direction, where HydABCD acts as an uptake hydrogenase, consuming H_2_ (21, 22).

**Fig 4:**
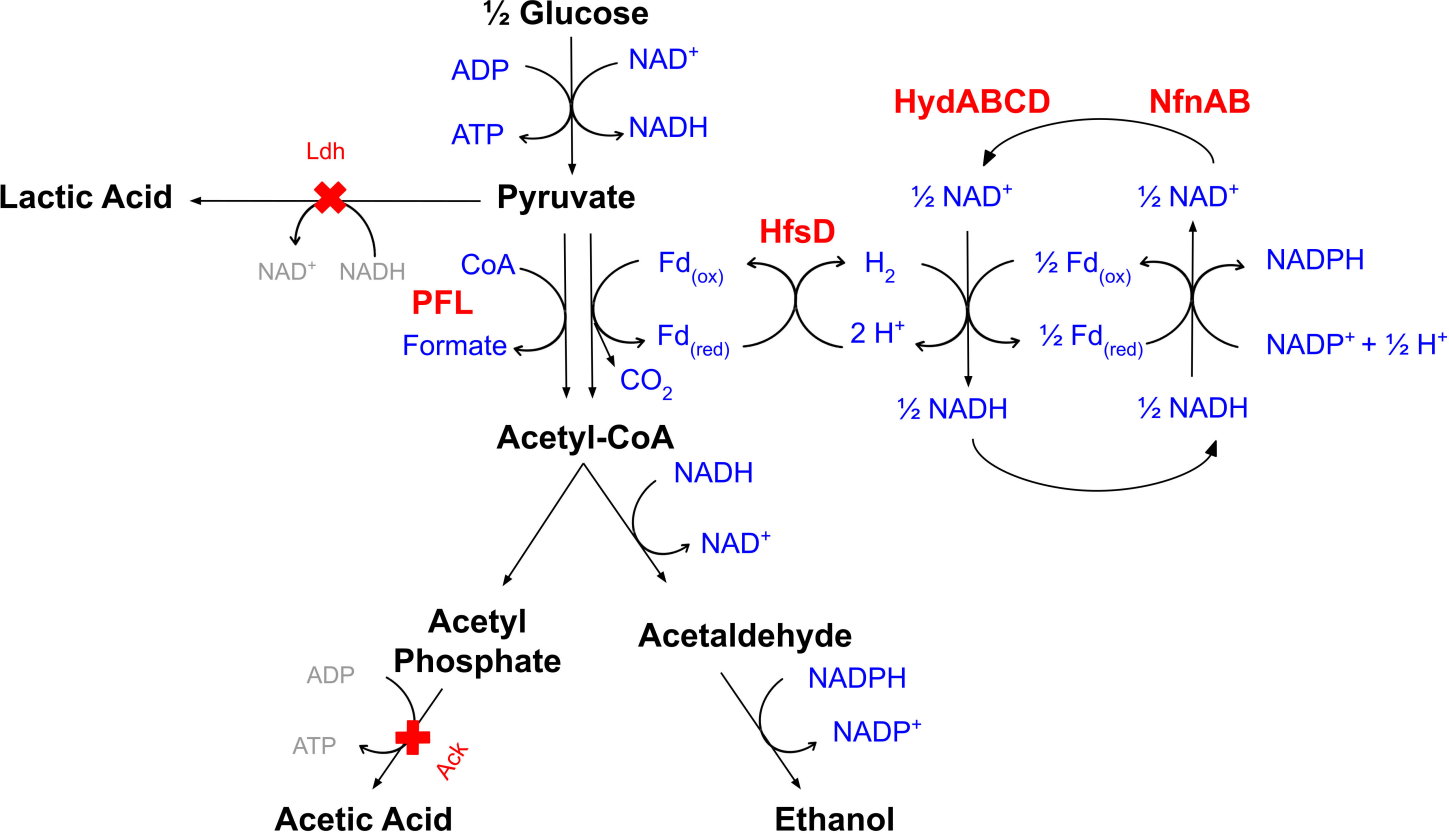
Hypothesized mechanism for ferredoxin re-oxidation in the context of ethanol production in *T. thermosaccharolyticum*. The proteins HfsD, HydAB, and NfnAB are hypothesized to catalyze the conversions shown, resulting in a net reaction equivalent to canonical FNOR.

With a stoichiometry equivalent to a canonical FNOR, we hypothesize the possibility of three different enzymes creating a reaction that would work as a functional FNOR through hydrogen cycling: HfsD, HydABCD, and NfnAB (Fig. 4).

In a scenario where the products of three gene clusters create a reaction similar to a FNOR reaction, our hypothesis of how they could be functionally linked is based on hydrogen cycling. Reduced ferredoxin (Fd_red_) would be utilized by HfsD to reduce protons to hydrogen. Meanwhile, the bifurcating hydrogenase (HydABCD) would produce NADH and reduced ferredoxin (Fd_red_) in the reaction. The third component in this trio, NfnAB, would consume the NADH and the reduced ferredoxin (Fd_red_) produced by HydABCD. Considering 1 mole of glucose, the reactions of each protein and the net reaction of the hypothesized reaction are shown below:


$$HfsD\;reaction:2\;Fd_{\left(red\right)}+3H^{+}+1H^{+}\to 2Fd_{\left(ox\right)}+2H_{2}$$

$$HydABCD\;reaction:2H_{2}+NAD^{+}+Fd_{\left(ox\right)}\to 3H^{+}+NADH+Fd_{\left(red\right)}$$

$$NfnAB\;reaction:Fd_{\left(red\right)}+NADH+2NADP^{+}+1H^{+}\to Fd_{\left(ox\right)}+NAD^{+}+2\;NADPH$$

$$Net\;reaction:2\;Fd_{\left(red\right)}+2\;NADP^{+}+2H^{+}\to 2\;Fd_{\left(ox\right)}+2\;NADPH$$

A possible mutation in the *hyd* operon could reduce its protein activity, and therefore, the amount of hydrogen reoxidation, leading to higher acetate and hydrogen production levels. At high hydrogen partial pressure, the combination of the hydrogen-consuming reaction of Hyd and the hydrogen-generating HfsD reaction promotes a stoichiometry equivalent to the FNOR reaction. With combined HfsD + HydABCD + NfnAB activity, it is possible to generate the stoichiometry of a functional NADPH-generating FNOR through H_2_ cycling. We do not exclude the possibility of protein-protein interactions in this context; however, such interactions are not totally required if hydrogen (H_2_) serves as the intermediary between the systems.

In *Thermococcus onnurineus NA1,* hydrogen was found to be an intermediate for NADP^+^ reduction. An association between a hydrogenase (*frhAGB-*encoded hydrogenase) and a formate dehydrogenase (Fdh3) results in the direct transfer of electrons between the proteins without the presence of an electron carrier. This suggests a coordinated electron transfer that may be crucial to redox equilibrium and energy metabolism in this species (23). We have not yet been able to determine how direct the association between HfsD and HydAB is; however, we believe that a similar coordinated electron transfer may be occurring.

Complementing previous work that demonstrated the deletion of *hfsB* as a technique to achieve ethanologen phenotypes (13), we show that the sole expression of *hfsD*, without any other gene from its operon, is enough to increase ethanol yields, as long as *hydAB* and *nfnAB* are present. The deletion of the entire *hfs* operon, followed by the overexpression of *hfsD,* resulted in a phenotypically stable mutant strain useful for subsequent genetic manipulations.

It is worthwhile to observe the patterns obtained between the expression of *hfsD* and formate production. Eminoglu et al. (13) observed the diversion of flux toward lactate when *hfsD* was absent and suggested that this could be a phenomenon caused by pyruvate overflow. This means that without *hfsD*, pyruvate is not efficiently converted into acetyl-CoA, possibly due to a deficient PFOR reaction. In the current study, where *ldh* is deleted, we speculate that the absence of *hfsD* leads to a diversion of flux toward the pyruvate formate lyase (PFL) reaction, an alternative reaction to oxidize pyruvate (pyruvate + CoA → acetyl-CoA + formate) (Fig. 4). In the absence of *hfsB* and the presence of a functional FNOR reaction, *T. thermosaccharolyticum* seems to favor the PFOR-FNOR reaction over the PFL reaction to convert pyruvate to acetyl-CoA.

Unfortunately, the results in the double mutant strains that include a deletion of *hfsB* gene are not conclusive due to the instability of the phenotype. A better understanding of the spontaneous mutations in the *hfsB* gene may be helpful to comprehend how to avoid or bypass the phenotypic reversion issue. Although phenotypic instability was encountered in strains LL1862, LL1865, and LL1866, steps were taken to mitigate it by finding alternatives for obtaining engineered ethanologen strains, as observed in strain LL1872. All other strains exhibited consistent and stable phenotypes.

We observed, for the first time, evidence that the *ech* operon is active in *T. thermosaccharolyticum*. It is possible that the elimination of other hydrogenases selected for its activation. After the deletion of the *ech* gene cluster, we obtained hydrogenase-minus strains (LL1868 and LL1869). The ability of these strains to still produce ethanol was intriguing since they do not contain two of the components hypothesized as responsible for the FNOR reaction. In a condition where they are absent, the microorganism may be able to adapt by using different cofactors or different components. The fact that the hydrogenase-minus strains showed growth defects in low concentrations of yeast extract leads us to consider that components of yeast extract may be supporting electron-balanced sugar metabolism in this strain.

We performed whole-genome sequence analysis on Δ*hfsB* strains that underwent independent laboratory evolution. Eighty percent of the strains showed mutations in the *hyd* operon. Interestingly, we observed independent recurrence in the affected genes among these strains. However, the relationship between mutations observed in strains RF1 and RF3 and the phenotypic reversion remains unclear. Despite lacking mutations in the *hyd* operon, these strains exhibited nucleotide alterations that were observed in other strains with a reverted phenotype. Although not fully understood, the sequence data suggest a consistent biological pattern linking the *hfs* operon with the *hyd* operon.

The contribution of this work is to document intriguing genetic results along with the presentation of a hypothesis of a hydrogen-cycling reaction equivalent to a FNOR reaction in *T. thermosaccharolyticum*. Identification of genes necessary for high ethanol yield suggests the presence of a complex and novel hypothetical pathway of Fd recycling. Further biochemical validation can potentially advance the efforts to engineer the cost-effective conversion of sugars to ethanol.

## MATERIALS AND METHODS

### Microorganisms

Work was performed with *T. thermosacchaloryticum* strain M0795, using a strain in which the genes *hpt* (encoding hypoxanthine phosphoribosyl transferase) and *ldh* (encoding lactate dehydrogenase) were eliminated with removable suicide plasmids constructed in *E. coli* as previously described (3, 24). Notably, the production of butyrate in *T. thermosaccharolyticum* is an important distinction between this strain and *T. saccharolyticum*. Butyrate production was eliminated in M0795 by the deletion of the gene encoding thiolase (*thl*). The resulting markerless strain LL1859 (*Δhpt Δldh Δthl*) was the parent strain for all subsequent experiments described here. Building upon this strain, further knockouts were performed following the steps explained in the following sections, resulting in 18 new strains (Table 5). *T. thermosaccharolyticum* is similar to *T. saccharolyticum* and also shows natural competence. Additionally, *T. thermosaccharolyticum* has an optimal pH similar to that of *C. thermocellum*, which creates the possibility of co-culturing these organisms.

**TABLE 5:** *T. thermosaccharolyticum* strains used in this work

Strain	Description	Genotype	Reference
M0795	*Δhpt Δldh*	*Δhpt Δldh*	Not published
LL1859	M0795 *Δhpt Δldh Δthl*	*Δhpt Δldh Δthl*	This work
LL1860	LL1859 *ΔhfsABCD*	*Δhpt Δldh Δthl Δ hfsABCD*	This work
LL1861	LL1859 *ΔhydAB*	*Δhpt Δldh Δthl ΔhydAB*	This work
LL1862	LL1859 *ΔhfsB*	*Δhpt Δldh Δthl ΔhfsB*	This work
LL1863	LL1860 *ΔhydAB*	*Δhpt Δldh Δthl ΔhfsABCD ΔhydAB*	This work
LL1864	LL1861 *ΔhfsABCD*	*Δhpt Δldh Δthl ΔhydAB ΔhfsABCD*	This work
LL1865	LL1862 *ΔhydAB*	*Δhpt Δldh Δthl ΔhfsB ΔhydAB*	This work
LL1866	LL1861 *ΔhfsB*	*Δhpt Δldh Δthl ΔhydAB ΔhfsB*	This work
LL1867	LL1859 *Δech*	*Δhpt Δldh Δthl Δech*	This work
LL1868	LL1863 *Δech*	*Δhpt Δldh Δthl ΔhfsABCD ΔhydAB Δech*	This work
LL1869	LL1864 *Δech*	*Δhpt Δldh Δthl ΔhydAB ΔhfsABCD Δech*	This work
LL1871	LL1860 *ΔnfnAB*	*Δhpt Δldh Δthl ΔhfsABCD ΔnfnAB*	This work
LL1872	LL1860 *+ hfsD*	*Δhpt Δldh Δthl ΔhfsABCD + hfsD(Ttherm*)	This work
LL1873	LL1863 *+ hfsD*	*Δhpt Δldh Δthl ΔhfsABCD Δ hydAB + hfsD(Ttherm*)	This work
LL1874	LL1864 *+ hfsD*	*Δhpt Δldh Δthl ΔhydAB ΔhfsABCD + hfsD(Ttherm*)	This work
LL1875	LL1863 *+ hydAB*	*Δhpt Δldh Δthl ΔhfsABCD ΔhydAB + hydAB(Ttherm*)	This work
LL1876	LL1864 *+ hydAB*	*Δhpt Δldh Δthl ΔhydAB ΔhfsABCD + hydAB(Ttherm*)	This work
LL1877	LL1865 *+ hydAB*	*Δhpt Δldh Δthl ΔhfsB ΔhydAB + hydAB(Ttherm*)	This work
LL1878	LL1866 *+ hydAB*	*Δhpt Δldh Δthl ΔhydAB ΔhfsB + hydAB(Ttherm*)	This work
LL1889	LL1871 *+ hfsD*	*Δhpt Δldh Δthl ΔhfsABCD ΔnfnAB + hfsD*	This work

### Media and cultivation conditions

Bacteria were cultured under anaerobic conditions at 55°C–60°C. *T. thermosaccharolyticum* was cultivated in M122C medium at pH 6.7 (25), whereas CTFUD media at pH 7.0 (26) was used for the transformation protocol and fermentation assays. To reduce the potential contribution of yeast extract as a carbon source, we tested different concentrations (Table S6) and chose a concentration of 0.5 g/L for fermentation assays with genetically modified strains, except for the hydrogenase-minus strains (*Δhfs Δhyd Δech;* LL1868 and LL1869), for which the usual concentration of 4.5 g/L of yeast extract was maintained.

### Plasmid construction

Plasmids were designed using the software CLC Main Workbench 22.0.2 (QIAGEN), then constructed using the method of Gibson (27) with the NEBuilder HiFi DNA Assembly Master Mix (New England Biolabs - NEB), and then purified and extracted with the Monarch Plasmid Miniprep Kit (NEB). In the suicide plasmids, used to perform genetic knockouts, the genes were targeted based on flanking homology arms, where the target gene stands in the middle of the upstream and downstream flanks. The overexpression plasmids were constructed using the backbone from pUC18 and the *cbp* promoter (Clo1313_1954) from *Clostridium thermocellum*. The plasmids were transformed into *T7 express E. coli* (NEB) using the appropriate antibiotic selection corresponding to the construct (kanamycin or carbenicillin). To confirm the correct construction of the plasmids, enzyme digestion and Illumina sequencing were performed.

### Genetic modification and transformation

The transformation of *T. thermossacharolyticum*, a species with natural competence, was performed as described in Shaw, Hogsett, and Lynd (7). The markerless knockouts in *T. thermosaccharolyticum* were performed according to Shaw et al. (24), with modifications. A 2-step plasmid-based gene replacement method was used, where the two steps are insertion of an antibiotic resistance gene and counterselection against the *tdk* gene (26). Kanamycin and 5-fluoro-2’-deoxyuridine (FUDR) were used as selection markers. Evidence of successful genetic modification is represented in Fig. S1.

### Serial transfer of *∆hfsB* mutants

The initial study of phenotype reversion was performed with a single isolate. From strain LL1859, a new mutant lacking the *hfsB* gene (strain LL1862) was obtained following the method described in the previous section. After confirmation of the deletion using PCR with external primers, the colonies were plated in M122C agar. A single colony was inoculated in 5 mL of M122C and a serial transfer of 5 µL to 4.95 mL of M122C medium was made. After 4 days, 50 µL of the cell culture was transferred to 50 mL of CTFUD overnight and prepared for whole-genome sequence analysis.

For the later analysis of the 10 new isolates, we re-generated the mutant strain lacking the *hfsB* gene, first by kanamycin selection for insertion/deletion and then by FUDR counterselection for removal of the Kan-tdk genes. From the FUDR counter selection plate, single colonies were picked into CTFUD medium. Half of the volume of these cultures was boiled and analyzed by PCR to verify gene removal. The other half was cultured for approximately 5 h, then plated on M122C agar without antibiotics. Eleven colonies were isolated and subjected to fermentation analysis to validate the ethanologen phenotype. Following confirmation, one colony was preserved at −80°C for use as an ethanologen control. The remaining 10 strains were re-plated, and from each plate, three colonies were picked. These clones were cultured independently and underwent 10 consecutive serial transfers. After a period of 10 days, the phenotype was evaluated of all 30 cultures using high-performance liquid chromatography (HPLC). All showed mixed acid fermentation products; hence, 50 µL of cell culture from each strain was inoculated into 50 mL of CTFUD medium and incubated overnight. The cultures were centrifuged to pellet the cells, in preparation for whole-genome sequencing. Following cell pelleting, one strain from each triplicate of every strain was selected to proceed with DNA preparation and sequencing, whereas the remaining pellets were stored.

### Whole-genome sequence analysis

The strains of this project were sequenced by the Center for Quantitative Biology (Hanover, NH, USA) using the Illumina short-read sequencing method. The treatment and analysis of resequencing data were as described in (5) using CLC Genomics (QIAGEN).

### Fermentation assays

For the fermentation assays in *T.thermossacharolyticum*, anaerobic culture tubes (Balch-type) sealed with blue butyl rubber stoppers were purged with a gas mix of 20% CO_2_ + 80% N_2_. CTFUD media pH 7.0 was used containing 5 g/L cellobiose and 0.5 g/L yeast extract (except hydrogenase-minus strains were fermented using a concentration of 4.5 g/L yeast extract). The tubes were filled with 5 mL of media, leaving 25 mL of headspace filled with the mixed gas. In total, 100 µL of cells were added to the medium. All the cultures were incubated in anaerobic conditions at 55°C–60°C without shaking and grown for 72 h (28). HPLC and gas chromatography methods followed as described previously (28). Residual substrates and liquid products were acidified and measured using HPLC with refractive index detection in an Aminex HPX-87H column (Bio-Rad Laboratories, USA). The molecular hydrogen production after fermentation was measured using gas chromatography with a HayeSep D packed column. Nitrogen was used as the carrier gas at a flow rate of 8.2 mL/min.

The “% Theoretical Maximum Yield of Ethanol”' values shown in Tables 1 and 3 were calculated as the percent of the concentration of ethanol after fermentation over the product of the concentration of sugar consumed during fermentation and the maximum theoretical ethanol yield of 0.51.

## Data Availability

Plasmid sequences for the plasmids pLC15 (*hfsABCD* deletion in M0795 *T. thermosaccharolyticum* strain), pLC16 (*hydAB* deletion in M0795 *T. thermosaccharolyticum* strain), pLC17 (*hfsB* deletion in M0795 *T. thermosaccharolyticum strain*), pLC27 (*nfnAB* deletion in M0795 *T. thermosaccharolyticum* strain), pLC29 (*ech* deletion in M0795 *T. thermosaccharolyticum* strain), pLC21 (*hfsD* gene expression in M0795 *T. thermosaccharolyticum* strain) and pLC22 (*hydAB* gene expression in M0795 *T. thermosaccharolyticum* strain) may be accessed within the GenBank database under accession numbers PQ629924, PQ629925, PQ629926, PQ629927, PQ629928, PQ629929, and PQ629930. Genome re-sequencing analysis presented in Table 4 (*hyd* operon mutations in the 10 strains exhibiting reverted phenotype [RF] after 10 serial transfers) was derived by comparison of Illumina short read data to the genome sequence of *T. thermosaccharolyticum* strain M0795 (accession number CP003066.1). The complete data set showing all identified sequence differences is given in Table S7.

## SUPPLEMENTAL MATERIAL

Fig. S1 aem.01109-24-s0001.pdf Agarose gel images confirming successful gene deletion or insertion.

Table S1 aem.01109-24-s0002.xlsx Fermentation profiles of Δ*hfsB* strain LL1862 before and after serial transfers and whole genome sequencing.

Table S2 aem.01109-24-s0003.xlsx Fermentation profiles of all strains used in this work.

Table S3 aem.01109-24-s0004.xlsx Fermentation product profiles of 10 Δ*hfsB* strains that underwent serial transfer and whole genome sequencing.

Table S4 aem.01109-24-s0005.xlsx Summary of mutations found in strains with reverted phenotype after serial transfer.

Table S5 aem.01109-24-s0006.xlsx Summary of the genes mutated after transfers.

Table S6 aem.01109-24-s0007.xlsx Effect of different concentrations of yeast extract on the fermentation profile of strains LL1859 and LL1863.

Table S7 aem.01109-24-s0008.xlsx Complete data set of sequence differences identified by re-sequencing analysis presented in Table 4.

ASM does not own the copyrights to Supplemental Material that may be linked to, or accessed through, an article. The authors have granted ASM a non-exclusive, world-wide license to publish the Supplemental Material files. Please contact the corresponding author directly for reuse.
