# Supplementary material for: Genetic investigation of hydrogenases in Thermoanaerobacterium thermosaccharolyticum suggests that redox balance via hydrogen cycling enables high ethanol yield
Source: Appl Environ Microbiol. 2025 Jan 10;91(2):e01109-24. doi: 10.1128/aem.01109-24 (PMC11837493; doi:10.1128/aem.01109-24)

Figure S1. Agarose gel images to confirm successful gene deletions and gene expression. Expected size for ***ΔhfsABCD* deletion** using external primers on Wild Type (WT) strains is 6.7kb, for a markerless strain 2.1kb. Expected size for ***ΔhydAB* deletion** using external primers on Wild Type (WT) strains is 5.6 kb, for a markerless strain 2kb. Expected size for ***ΔhfsB* deletion** using external primers on Wild Type (WT) strains is 3.5 kb, for a markerless strain 2.1kb. Expected size for ***Δech* deletion** using external primers on Wild Type (WT) strains is 10.5 kb, for a markerless strain 2.1kb. Expected size for ***ΔnfnAB* deletion** using external primers on Wild Type (WT) strains is 4.3 kb, for a markerless strain 2.2kb. Expected size for the presence of the ***hfsD* gene** is 1.5 kb. Expected size for the presence of the ***hydAB* genes** is 3.5 kb.

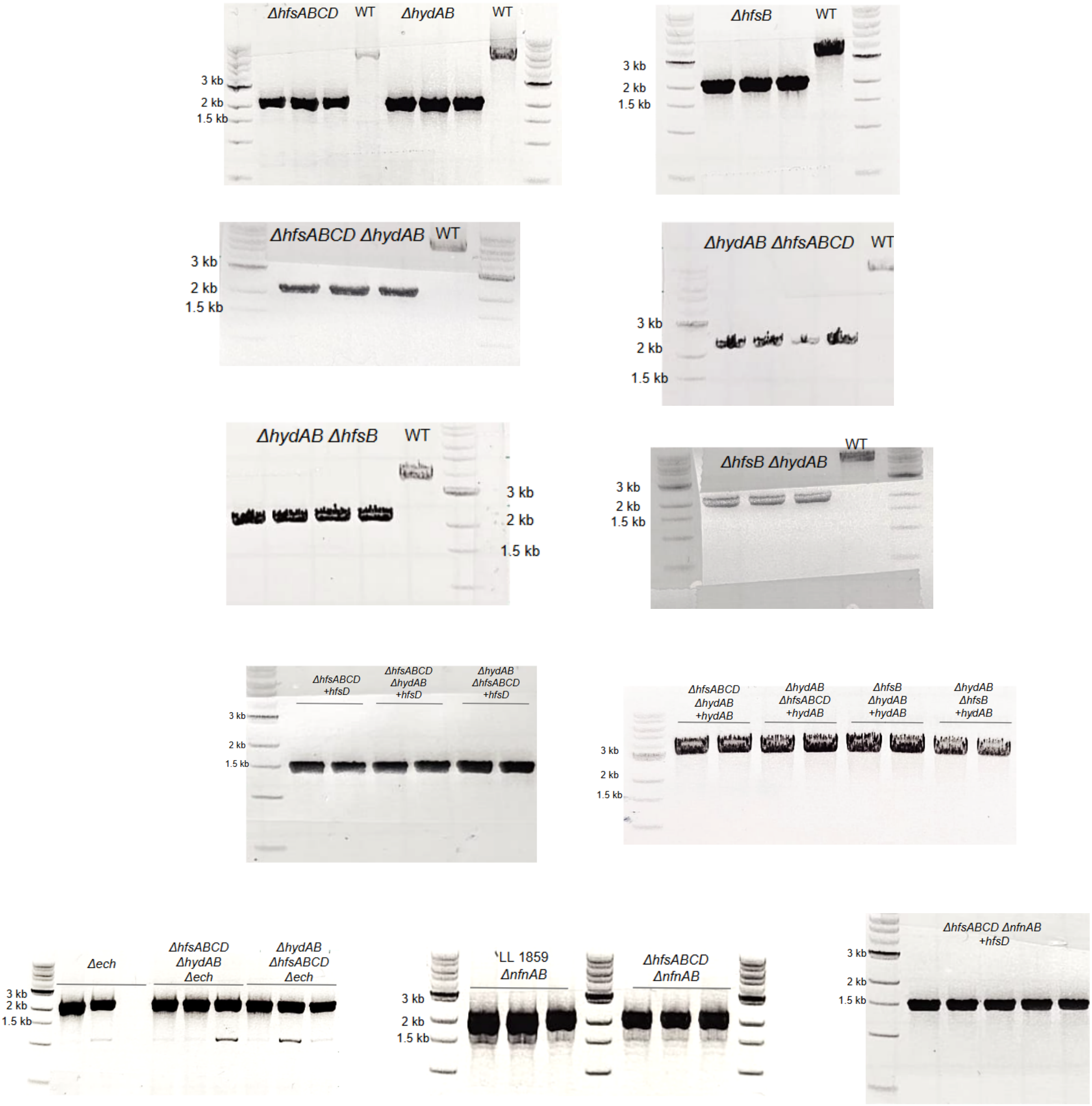

Supplement: Fig. S1 — Agarose gel images confirming successful gene deletion or insertion. [file aem.01109-24-s0001.pdf]
